# Inosine-Induced Base Pairing Diversity during Reverse Transcription

**DOI:** 10.1021/acschembio.3c00555

**Published:** 2024-01-22

**Authors:** Ya Ying Zheng, Kaalak Reddy, Sweta Vangaveti, Jia Sheng

**Affiliations:** † Department of Chemistry, University at Albany, State University of New York, 1400 Washington Avenue, Albany, New York 12222, United States; ‡ The RNA Institute, University at Albany, State University of New York, 1400 Washington Avenue, Albany, New York 12222, United States

## Abstract

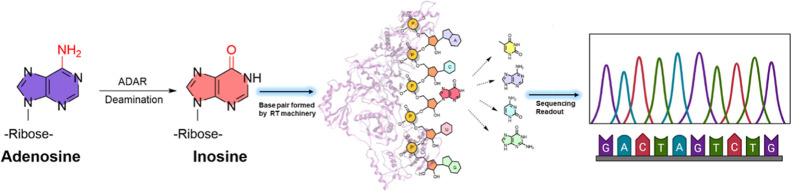

A-to-I editing catalyzed by adenosine deaminase acting on RNAs impacts numerous physiological and biochemical processes that are essential for cellular functions and is a big contributor to the infectivity of certain RNA viruses. The outcome of this deamination leads to changes in the eukaryotic transcriptome functionally resembling A–G transitions since inosine preferentially pairs with cytosine. Moreover, hyper-editing or multiple A to G transitions in clusters were detected in measles virus. Inosine modifications either directly on viral RNA or on cellular RNA can have antiviral or pro-viral repercussions. While many of the significant roles of inosine in cellular RNAs are well understood, the effects of hyper-editing of A to I on viral polymerase activity during RNA replication remain elusive. Moreover, biological strategies such as molecular cloning and RNA-seq for transcriptomic interrogation rely on RT-polymerase chain reaction with little to no emphasis placed on the first step, reverse transcription, which may reshape the sequencing results when hypermodification is present. In this study, we systematically explore the influence of inosine modification, varying the number and position of inosines, on decoding outcomes using three different reverse transcriptases (RTs) followed by standard Sanger sequencing. We find that inosine alone or in clusters can differentially affect the RT activity. To gain structural insights into the accommodation of inosine in the polymerase site of HIV-1 reverse transcriptase (HIV-1-RT) and how this structural context affects the base pairing rules for inosine, we performed molecular dynamics simulations of the HIV-1-RT. The simulations highlight the importance of the protein-nucleotide interaction as a critical factor in deciphering the base pairing behavior of inosine clusters. This effort sets the groundwork for decrypting the physiological significance of inosine and linking the fidelity of reverse transcriptase and the possible diverse transcription outcomes of cellular RNAs and/or viral RNAs where hyper-edited inosines are present in the transcripts.

## Introduction

Post-transcriptional modifications are essential for the proper function and fine-tuning of all classes of RNAs. To date, more than 170 chemical modifications have been identified in both coding and noncoding RNAs, adding an extra layer of complexity to transcriptome regulation.^1^ These modifications have been shown to play an important role in modulating various cellular and biological processes including RNA metabolism, structural stability, splicing, transport, and signaling pathways.^2,3^ RNA editing by means of C6-hydrolytic deamination of adenosine to inosine is prevalent in tRNAs, mRNAs, miRNAs, as well as some viral RNAs.^4−7^ Conventional methods for inosine detection rely on reverse transcription and polymerase chain reaction (RT-PCR) where the change in the cDNA is compared with its corresponding genomic sequence with A to G replacement (A to G is observed since I preferentially pairs with C and results in G readout).^8^ Several more advanced biochemical strategies have been proposed to accurately identify inosine editing sites. Specifically, inosine chemical erasing (ICE) is based on the termination of cDNA synthesis at the site of inosine modification when inosine is cyanoethylated;^9^ nanopore RNA seq relies on the differences in the rate of current flow for each modification;^10^ endonuclease-mediated seq involves the blocking of 3′-OH of RNA, followed by hEndoV cleavage at inosine sites to generate a new terminal 3′OH for direct sequencing analysis;^11^ lastly, an inosine derivatization strategy coupled with LC-MS/MS analysis was reported to drastically improve its detection sensitivity.^12^

Depending on the RNA type, inosine can exert different functional activities. Initially discovered in tRNA in 1965,^13^ inosine at the wobble position in the anticodon stem loop, I_34_, can profoundly diversify codon recognition and potentiate the flexibility of translating U-, A-, and C-ending codons compared to A_34_ which recognizes only U-ending codons.^14−16^ A-to-I editing in mRNA is catalyzed by the adenosine deaminase acting on the RNA (ADAR) enzyme family.^17^ Inosine in the transcripts can affect pre-mRNA splicing, microRNA silencing, RNA stability, and altering interactions with RNA binding proteins. mRNA with excess inosine misincorporation has been shown to induce translational stalling^18^ and can even lead to the truncation of the peptides.^19^ Moreover, depending on the number of inosines, especially at the ends of codon triplets III, IAI, ICI, or IUI, translation can result in increased truncation rates of about 30% compared to 5% for single modifications.^19^ Deregulated A-to-I editing has been implicated to play a role in human diseases including neurological disorders, defects in signaling pathways and cancers.^20−23^ Selectively editing specific miRNA precursors can reprogram the miRNA functionality,^24,25^ supported by the identification of approximately 16% of UAG triplets in human pre-miRNA that underwent A-to-I editing, which may result in the degradation of different target mRNAs.^26^ Studying the deamination on viral RNAs, specifically the lymphocytic choriomeningitis virus, showed that ADAR1-mediated hyper-editing of A to I led to nonfunctional viral glycoprotein at high frequency, indication of antiviral property.^27^ In addition, editing of the viral genome of both hepatitis C and polyomavirus attenuated the virulence of infection. In contrast, editing in other viruses including influenza, measles, and HIV exhibited a pro-viral property as it enhances viral proliferation.^28^ More recent investigation has also reported A-G mutation in SARS-CoV-2 viral sequence with more processive infectivity.^29,30^ While the presence of modifications in viral transcriptomes has long been documented,^31,32^ the transcription of biased or hyper-edited A to I remains largely unexplored and requires close scrutiny since inosine can either enhance or reduce viral infectivity.

Furthermore, reverse transcription by viral enzymes from a mRNA template is an essential step in molecular cloning for the expression of recombinant protein, interrogation of transcriptomic profiles in bulk or single cells, and in high-throughput sequencing or RNA-seq. RT-PCR is a critical tool in basic virology and biology research for RNA to DNA conversion. Hence understanding the effects of inosine modification in reverse transcription is of significance in physiological applications. Despite advancements in sequencing technology from next generation sequencing to single cell seq, the catalytic fidelity of reverse transcriptase used for reverse-transcribing noncanonical bases such as inosine is not well understood. Two enzymes—the reverse transcriptases of Moloney murine leukemia virus (MMLv) and avian myeloblastosis virus (AMV) are used extensively for cDNA synthesis due to their high efficiency and fidelity,^33^ while human immunodeficiency virus 1 (HIV-1) reverse transcriptase is rarely used considering its high error rate during DNA synthesis.^34^ The reported average error rates of misincorporation per nucleotide for these enzymes are ∼1/17,000, ∼ 1/30,000, and 1/1700 for AMV, MMLv, and HIV-1, respectively.^35^ Another study conducted in M13 bacteriophage revealed that the mutation frequencies for MMLv and AMV RTs were in the range of 3.3–5.9 × 10^–4^ errors/base, while HIV-1-RT was 5.9 × 10^–3^ errors/base. Moreover, among the three common errors introduced during DNA synthesis, the rate of substitutions was much higher than insertions or deletions.^33^

**Figure 1:**
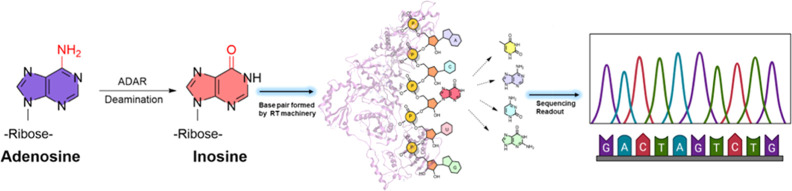
Formation and readout of inosine. The enzyme ADAR catalyzes the formation of inosine. Reverse transcription of inosine containing RNAs by enzymes is explored in a context-dependent manner. The preferential base pairing with inosine is investigated via the conventional Sanger sequencing methodology.

In this study, we use a combination of biochemical and computational techniques to explore the effect of inosine substitutions on the cDNA synthesis step in RT-PCR experiments. We used three reverse transcriptases—MMLv, AMV, and HIV-1 and a set of inosine containing RNA sequences to investigate the effects of varying the position and frequency of inosines on the cDNA readout (Figure 1).

## Results and Discussion

### Reverse Transcription of Inosine in Primer Extension Studies

**Figure 2:**
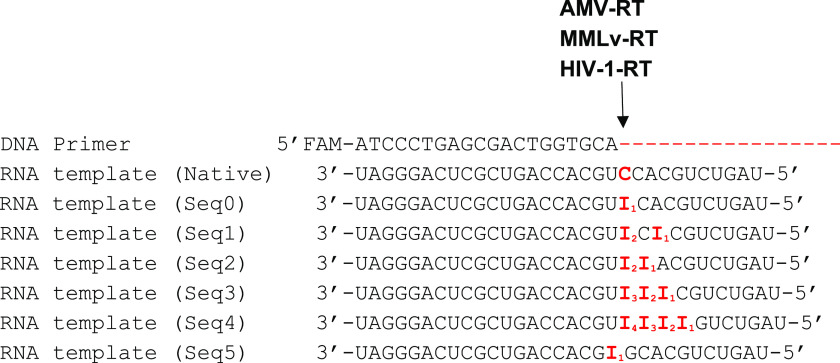
Inosine-modified RNA templates for sequence-directed primer extension. The reverse transcription reaction was carried out using three RTs: AMV, MMLv, and HIV-1 for the synthesis of complementary DNA (cDNA).

**Figure 3:**
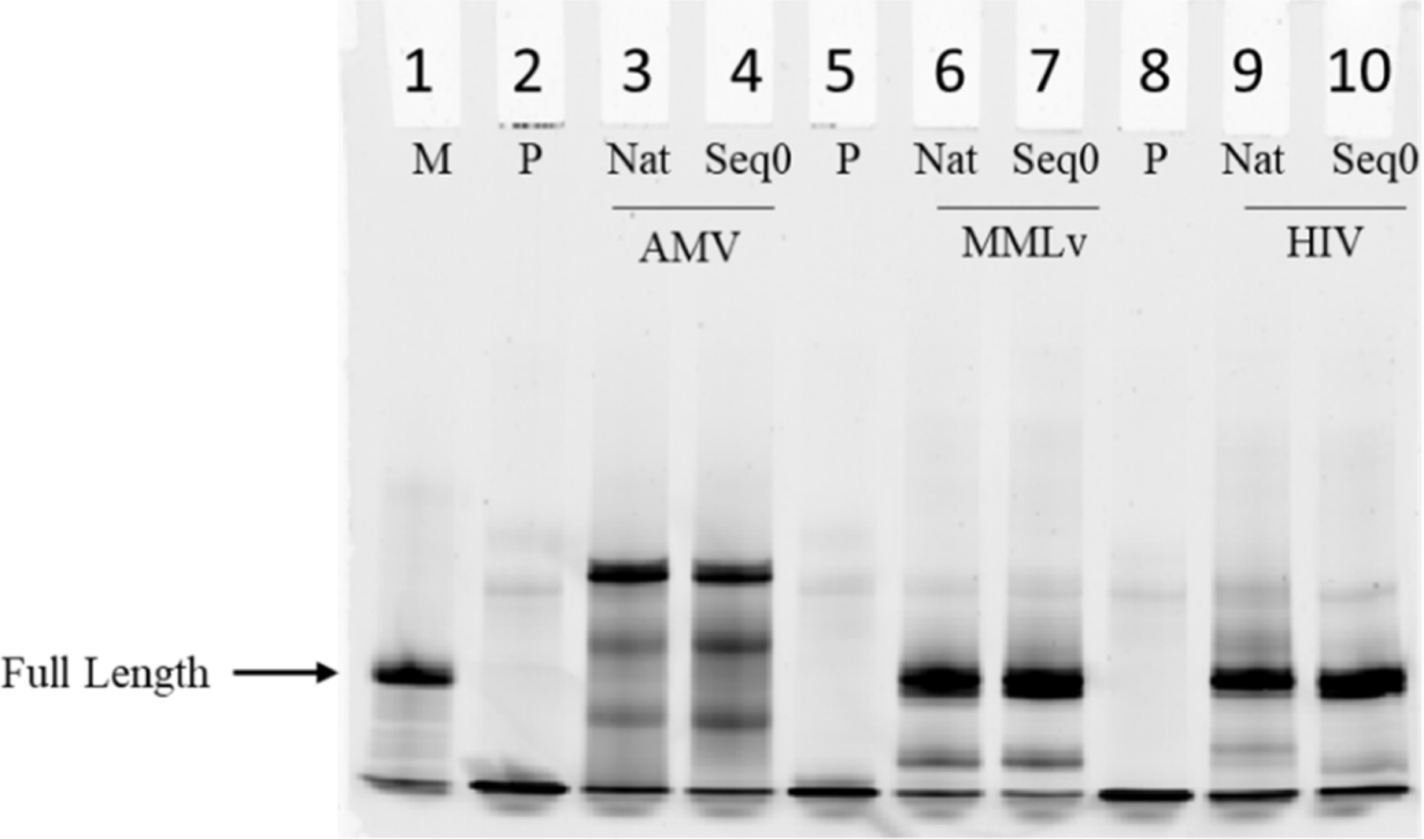
Fluorescent image of primer extension comparing three RTs for native and inosine-containing Seq0. From left to right, 30bp FAM-labeled RNA oligo was used as standard, followed by FAM-labeled primer, native strand, and Seq0 with single inosine modification as template for AMV, MMLv, and HIV-1. Samples were prepared under denaturing conditions and resolved on a 15% urea polyacrylamide gel electrophoresis (PAGE) gel. The image is obtained by using a typhoon scanner with the emission filter set to 520bp. All RTs could extend the templates for both native and inosine with AMV showing the most compelling result as the major product extended beyond the full length.

We began our investigation by first synthesizing native 3′-UAGGGACUCGCUGACCACGU**C**CACGUCUGAU-5′ and single inosine modified 3′-UAGGGACUCGCUGACCACGU**I**_1_CACGUCUGAU-5′ (Seq0) RNA strands, which our lab previously used to study reverse transcription activity,^36,37^ using an in-house solid-phase oligo synthesizer (Figure 2). We conducted RNA template directed primer extension reactions employing three different reverse transcriptases, namely, AMV, MMLv, and HIV-1. In this reverse transcription model, a DNA primer, labeled with a fluorescent FAM group at the 5′-end, consists of 20 nt preceding the inosine edited site. This reaction scheme allowed for comparison of the RT enzyme activity on inosine-containing RNA templates at the start of DNA polymerization. The full-length fluorescent products were then visualized by using a Typhoon scanner. It is widely acknowledged that both AMV and MMLv are extensively used in reverse transcription and RNA sequencing, with higher efficiency and fidelity compared to HIV. During reverse transcription of native and Seq0 sequences using AMV-RT, the reaction not only completed the base incorporation directed by the RNA template, but also extended beyond the full length (Figure 3, lanes 3 and 4). Three different lengths were observed for AMV-RT, with a dominant overelongated product (Figure 3, lanes 3 and 4). In contrast, MMLv and HIV-1 had no such overextension, and the major products were all of the expected length (Figure 3, lanes 6 and 7 and 9 and 10). Taken together, these observations suggest that a single inosine substitution in the RNA strand does not perturb the activity of the reverse transcription with respect to the length of the DNA product since both native and Seq0 had similar results for all three RTs.

**Figure 4:**
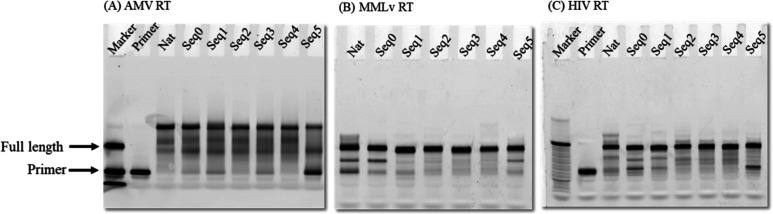
Fluorescent images of primer extension using AMV, MMLv, and HIV-1 RTs for all templates. (A) FAM-labeled RNA oligo 30bp, 20bp, and 10bp in length used as standard and negative control with all components in the reaction but template, native strand, and the inosine modified templates from Seq0 to Seq5 for AMV-RT. (B) Native followed by Seq0-Seq5 for MMLv-RT (C) 30bp FAM labeled RNA standard followed by negative control, native, and Seq0-Seq5 for HIV-1-RT. The denatured samples were resolved in a 15% urea PAGE gel. The image was obtained by using a typhoon scanner with the emission filter set to 520BP. While all the RTs extended the templates, only AMV showed a significant over extension as compared to the other two RTs.

Next, we asked whether multiple inosine sites would alter the reverse transcription outcome. To explore this, we synthesized several RNA templates with varying positions and frequency of inosines as substrates in the system. Specifically, we synthesized five additional RNA strands Seq1-Seq5 that differed in the number and position of inosine sites (Figure 2). These 31nt-long modified RNAs all have inosine at the polymerization start site except Seq5 where the inosine site overlaps with the primer to serve as a control. The enzymatic activity of reverse transcription of the inosine modified RNAs was analyzed via our primer extension model and resolved on 15% denaturing urea gels. The results were surprisingly similar to those of the singly substituted inosine sequence Seq0. AMV-RT consistently produced cDNA longer than the expected length based on the substrate RNA strand. To examine whether the RNA directed DNA product is truly longer than the template for AMV-RT, we used a marker containing 10, 20, and 30 nt. The resulting gel showed that all of the products were extended beyond the full lengths, implying that the complexity of inosine modification does not have an influential role in the reaction. We speculate that the initial enzyme binding and recognition may attribute to the over extension product of the reaction (Figure 4A). On the other hand, both MMLv and HIV-1 RTs showed full length products for all RNAs independent of the modification state of the substrate. This indicated that the presence of single or multiple clustered inosines in the given transcript has no effect on the length of cDNA generated when using MMLv and HIV-1 reverse transcriptases (Figure 4B,C), suggesting that these enzymes can efficiently reverse transcribe RNA templates with varying inosine contents.

### Base Readout for Inosine-Modified RNAs via Sanger Sequencing

**Figure 5:**
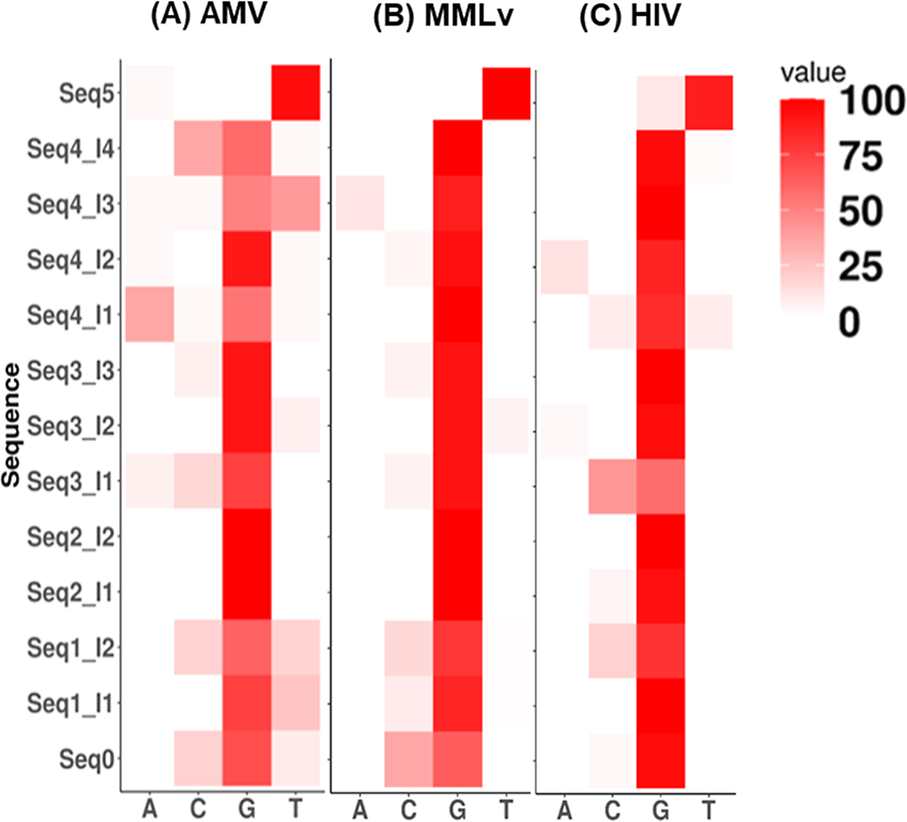
Heatmaps showing the base percentage of inosine readout. Sequencing results of AMV-RT (A), MMLv-RT (B), and HIV-1-RT (C) reverse transcription with inosine-modified strands. The color intensity in the heatmap represented the likelihood of a given nucleotide A, C, G, and T being read in the place of inosine modification.

While the gel-based assay detects the length of the cDNA product as a result of reverse transcription of modified RNA strands, it does not show the complementary nucleotides. So, we employed standard Sanger sequencing to investigate the specific nucleotide incorporation opposite to inosine. Since PCR fragments are usually required to be at least 300-bp to overcome poor quality reads from the beginning of the primer binding site, we could not directly sequence the resulting cDNA from our previously designed reverse transcription experiments with 31bp RNA substrates. To overcome this limitation, we first lengthened our cDNA with poly A tail extension, followed by PCR amplification and finally cloned the DNA into plasmids for sequencing (Figure S1). Approximately 70 clones were picked for the analysis of inosine base complementarity in relation to the fidelity of reverse transcription by each representative enzyme. Among the total clones picked, the ratio of sense versus antisense inserts were about the same, which is a recognized disadvantage of TA cloning, where insertion is nondirectionalized.^38^ Our combined results were depicted in a heatmap (Figure 5) with the results also available as a corresponding sequence logo in Supporting Information (Table S1). All of the antisense strands were converted to their complementary sense strands prior to analysis. The color intensity in the heatmap represents the likelihood of a given nucleotide A, C, G, and T being read in the place of the inosine modification. Several interesting findings were evident from the heatmap. As expected, G is the most dominant readout in most cases, implying that the I/C pair is the most preferred one. Meanwhile, I/C, I/T, and I/A are possible base-pairs that inosine can form, the possibility of the forming pairs is enzyme and sequence context-dependent. In Seq1, where two inosines are present at alternate positions, it is interesting to note that for all the three RTs (Figure 5A–C), a C readout is frequently observed for the I_2_ residue in Seq1, indicating that an I/G mispair could be well tolerated in this context. Seq2, Seq3, and Seq4 have two, three, and four consecutive inosines in the sequences, respectively. Seq2 with two inosines together has expected behavior with a G dominant readout for both positions, implying an I/C pair at the cDNA synthesis step. In Seq3, the I_1_ residue has C as the second dominant readout after G for all three RTs, similar to that observed in Seq1. Seq3 and Seq4 have variability in the readouts among the three RTs. Notably, while HIV-1-RT is considered the most error prone RT among the three enzymes, AMV-RT seems to have the most variability when interacting with inosine. Another interesting observation is the nearly absent or faint A-readout which indicates a weak or absent I/T pair. Based on our result, the relative base pairing preference of inosine is (I/C > I/G > I/A > I/T, resulting in a G > C > *T* > A read out), which differs from the conventional ranking of inosine base pairing (I/C > I/*T* > I/A > I/G, resulting in a G > A > *T* > C read out) (Figure 5).

### UV-Thermal Melting Temperature *T*_m_ Study

**Figure 6:**
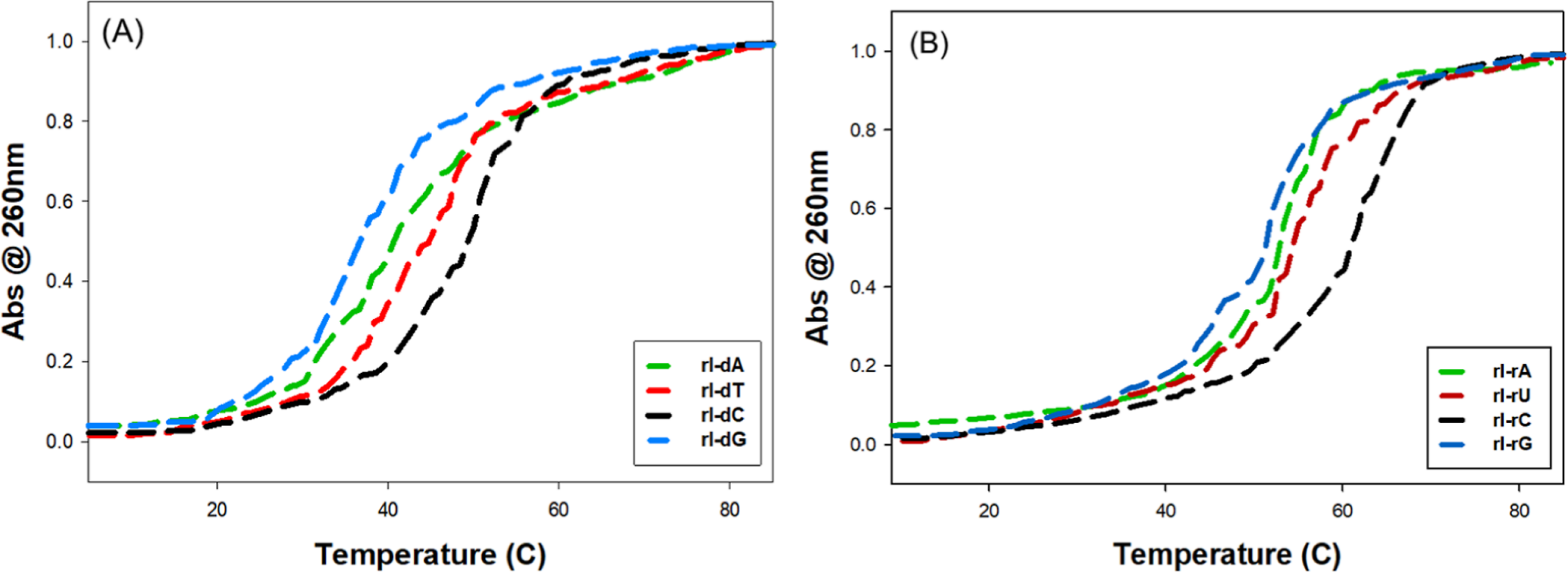
Normalized UV-melting curves of inosine-containing RNA duplexes. (A) 12nt inosine matched with complementary DNA strand with a single nucleotide variation of A,T,C,G paired at the site of modification. (B) 12mer inosine matched with a complementary RNA strand with a single nucleotide variation of A,U,C,G paired at the site of modification.

Most of what is known about the base-pairing preferences of inosine comes from RNA/RNA duplex studies of poly inosines or in the context of codon/anticodon interactions.^39^ To further explore the preferential base pairing of inosine in an RNA/DNA hybrid, we employed thermodynamic melting studies of the duplexes containing 12 nt inosine modified RNA (5′-AAU GC**I** GCA CUG- 3′) and its complementary DNA and RNA counterparts (5′ CAG TGC **dX**GC ATT 3′) and (5′-CAG UGC **rX**GC AUU-3′), respectively, where X is A, T/U, C, or G as explained in the methods. Compared to genomic DNA, DNA/RNA hybrids are indispensable biological intermediates form during replication and reverse transcription.^40,41^ The formation of duplex RNAs is crucial in maintaining cellular homeostasis as in naturally occurring interference RNA (RNAi).^42,43^ The normalized *T*_m_ curves are depicted in Figure 6 with detailed thermodynamic parameters in Table 1. Our result showed higher thermal stability in duplex RNA than DNA/RNA hybrid, which is consistent with other reported studies.^41,44^ This can be attributed to the presence of an additional 2′-OH group that positioned duplex RNAs in a stabilized A form with an extended water network of hydration effect along its minor groove.^41,45,46^ The inosine base pair hierarchy is similar in both RNA duplexes and RNA/DNA hybrid. We observe the stability trend that is consistent with past reported studies of I/C > I/T/U > I/A > I/G for both DNA and RNA where I/T or I/U shows a higher thermal stability than I/A. The difference in the *T*_m_ of rI/dC and rI/dG is 12.56 °C with a reduction in Δ*G* of 3.67 kcal/mol. Similarly, the difference in the *T*_m_ for rI/rC and rI/rG is 9.64 °C with an Δ*G* of 1.98 kcal/mol. While these results explain the dominant G-readout in our sequencing data, they do not provide any hints into the tolerance of I/G base-pairs, leading to a C-readout in our sequenced data.

**Table 1:** Thermodynamic Measurements of Inosine-Modified RNA in Duplexes^a^

base pair	*T*_m_ (°C)	–Δ*G* (kcal/mol)	Δ*H* (kcal/mol)	Δ*S* (cal/kmol)
rI/dA	38.1	7.6	58.4	163.7
rI/dT	42.2	8.6	72.6	206.1
rI/dC	48.3	10.8	93.7	267.1
rI/dG	35.8	7.1	65.8	189.2
rI/rA	52.4	11.8	93.0	261.6
rI/rU	53.6	12.8	103.9	293.8
rI/rC	61.0	13.9	90.4	246.7
rI/rG	51.3	11.9	101.0	287.1

a 1.5 μM 12 nt inosine RNA annealed with matched 1.5 μM DNA or RNA strand in a total volume of 600 μL of sodium phosphate buffer. The thermodynamic values reported here are the average measurements of four ramps.

### Molecular Dynamics Simulations

**Figure 7:**
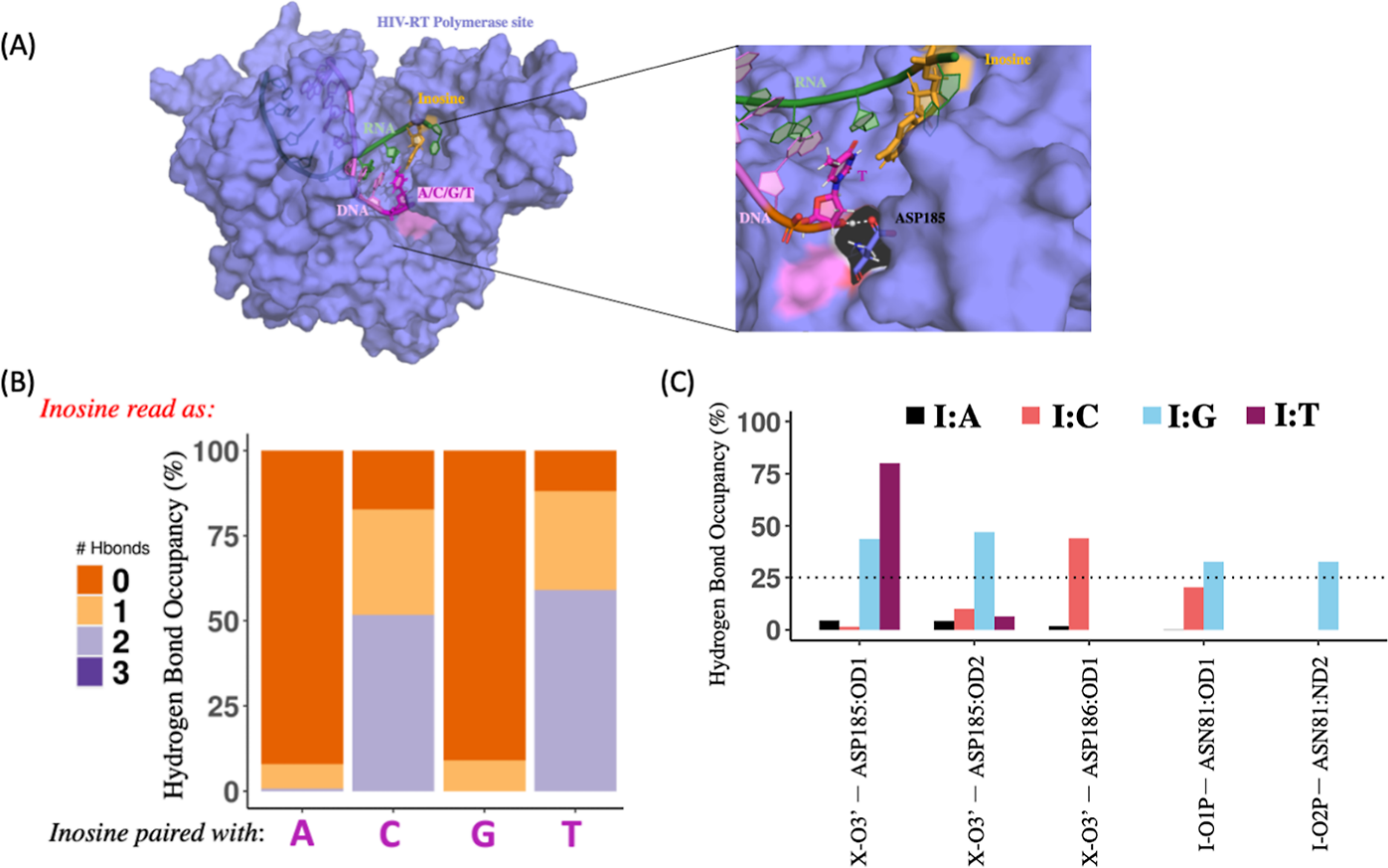
Molecular dynamics simulations of the HIV-1-RT polymerase site with inosine in the RNA substrate. (A) Structure of the simulated HIV-1-RT in purple, with the RNA substrate in green, and the DNA product strand in pink. Inosine is highlighted in orange. (B) Percent hydrogen bond occupancy for base-pairing patterns of inosine—the percentage of the simulation time that I: (A/C/G/T) pairs form 0,1, 2, or 3 hydrogen bonds. (C) Percent hydrogen occupancy for interaction of the inosine containing base pair with the RT—the percentage of the simulation time that the I: X (A/C/G/T) forms a hydrogen-bond with the surrounding amino acids in the RT at the polymerase site.

Since HIV-1-RT showed less variability based on our heatmap and as one of the most well documented enzymes, we chose this enzyme to gain insights into how different nucleotides are accommodated opposite inosine by the RTs. We performed molecular dynamics simulations of the polymerase site of HIV-1-RT, with inosine at the active site of the RNA substrate and A,C,G,T base-paired with I on the DNA product (Figure 7A). To assess the base-pairing preference, we calculated the hydrogen bonding patterns of I in the DNA/RNA hybrid in the context of enzyme. In our simulations, I/Y (C/T) base pairs form two hydrogen bonds for at least 50% of the simulations, while no hydrogen bonds are observed for the I/R (A/G) pairing. This suggests that even though it is possible for an I: A pair to form in duplexes, the enzyme restricts purine/purine pairing at the polymerase site. This is reflected by the almost complete lack of T readout in our sequencing results for the HIV-1-RT (Figure 5C). However, I/G pairing is observed frequently as a C read out in our sequencing results. To understand this surprising result, we further analyzed the protein-nucleotide interactions at the polymerase active site in our simulations.

While base-pairing is important, the interaction of the newly formed hybrid pair with the protein also plays a critical role in nucleotide incorporation. All protein-nucleotide interactions we observed in our simulation are with the backbone of the DNA/RNA hybrid. We find that the H-bond interaction with Asp186 is unique to the I/C pair, suggesting that it is important for nucleotide incorporation since the I/C pair has the strongest preference as noted in our sequencing and simulation results. A second interaction with Asn81 is observed for both the I/C and I/G pairs. While I/A pairs do not show any interactions with the surrounding protein, I/T has a strong interaction with Asp185, which it shares with the I/G pair as well. In summary, the hydrogen bonds analysis for the I/X pairs and their interaction with the surrounding protein in the polymerase site of HIV-1-RT suggest that (i) I/C is the most preferred pair. (ii) The I/T pair can form stably with two H-bonds, but its interactions with RT may not support its incorporation. (iii) The I/G pair does not form stable H-bonds but it can form an I/C like interaction with the surrounding polymerase, supporting incorporation. (iv) The I/A pair neither forms stable H-bonds nor does it have any interactions with the amino acid to support incorporation.

## Conclusions

In this study, we focused on the influence of single versus multiple inosine sites on base incorporation using three representative RT enzymes. Our results indicated that all three RTs extended the primer to full length, and the complexity of the inosine modified templates had little effect on reverse transcription activity with respect to the length of the products. Notably, AMV-RT showed more potent nontemplated terminal transferase activity and resulted in the extension of a couple of nucleotides longer than expected products for all RNA sequences, as shown in (Figure 4A). This is not observed for MMLv (Figure 4B) and HIV-1 (Figure 4C) despite also having such activity. Moreover, both AMV and HIV-1 RTs showed a decrease in productivity for Seq5 containing one base mismatch in comparison to MMLv.

The findings from the heatmap are interesting as we observed inosine decoding by AMV-RT showed C readouts, indicating potential I/G pairs formed when having two inosines in close proximity as in Seq1_I_2_, with three inosines as in Seq3; and four clustered inosines in Seq4, except on the second position (Figure 5A). By contrast, MMLv-RT showed a C readout for the second inosine in Seq4 that was absent in AMV-RT (Figure 5B). Lastly, HIV-1-RT showed C readout for almost all first positions apart from Seq1 consistent with AMV-RT Seq1_I_1_ with two inosines (Figure 5C). Albeit of less use in high-throughput sequencing, HIV-1-RT from the heatmap displayed the least disrupted reverse transcription of multiple inosines compared to RT enzymes most extensively used for cDNA synthesis, namely, AMV and MMLv. Although C readouts resulting from I/G pairs formed during reverse transcription are unstable in comparison to other base pairs, we showed that inosine could perhaps reshape coding properties by altering base discrimination based on the sequence context and presence in clusters, thereby altering the reverse transcription outcome. Moreover, the decoding properties of different RT enzymes may also contribute to the final sequence of cDNAs synthesized from inosine-containing RNA.

The stability of inosine base pairing was further examined by looking into the thermodynamic profiles using a thermal denaturation melting study (Figure 6). The duplex RNA with its matched DNA or RNA counterparts showed a hierarchy of stability decreasing from I/C > I/T/U > I/A > I/G, consistent with the known factor about base pairing behavior of inosine. We concluded the study by looking into the dynamic interactions of inosine modified RNA substrates in the polymerase active site of HIV-1-RT. The molecular simulation results revealed that protein interaction may be equally important as base pairing preference, as evidenced by fewer A readout compared to C readouts. This work provides some mechanistic insights into the HIV-1-RT polymerase active site on the decoding properties of multiple inosine substrate and potential miscoding of RNA in the case of hyper-edited sequences.

## Experimental Section

### Primer Extension

Primer extension was performed by the means of reverse transcription using three different reverse transcriptases, namely, AMV-RT (NEB), MMLv-RT (NEB), and HIV-1-RT (AS ONE Corp). Descriptive reaction protocol: reverse transcription was carried out in 20 μL total reaction volume containing 1X reverse transcription buffer: 50 mM tris-acetate, 75 mM potassium acetate and 8 mM magnesium acetate at pH 8.3 for AMV-RT, and 50 mM tris–HCl, 75 mM KCl and 3 mM MgCl_2_ at pH 8.3 for both MMLv and HIV-1-RTs. The final concentration of each reagent was as follows: RNA template 5 μM, FAM labeled DNA primer 2.5 μM, dNTP 0.5 mM, RNase inhibitor 20U and lastly each enzyme, AMV-RT 5U, MMLv-RT 100U, and HIV-1-RT 4U. The reagents were combined in 200 μL PCR tube and incubated in a thermocycler at 37 °C for 1 h and subsequently quenched by adding stop solution containing 98% formamide, 0.05% xylene cyanol, and 0.05% bromophenol blue, heated at 95 °C for 5 min, and cooled down on ice prior to resolving on a 15% PAGE 8 M urea gel at 250 V for 45 min. Typhoo imager was used to visualize the gel image.

### Synthesis of Both Native and Inosine Containing RNA Template Strands

The RNA templates used in the study were chemically synthesized at 1.0 μM scale using the Oligo-800 DNA/RNA solid-phase synthesizer. All anhydrous reagents were purchased from the ChemGenes Corporation. The operating system is protected under helium gas in the DMTr-off mode. This automated synthesis proceeds in the 3′ to 5′ direction and adds one nucleotide at the completion of four main steps detritylation, coupling, capping, and oxidation per synthesis cycle. Synthesis takes place on the control-pore glass (CPG-1000) immobilized with the first nucleotide through a succinate linker inside the column. Inosine phosphoramidite was dissolved in anhydrous acetonitrile to a concentration of 0.1 M. Trichloroacetic acid in methylene chloride (3%) was used for the 5′-detritylation or the removal of 5′DMT protecting group on the 3′end of oligonucleotide. Coupling was carried out using 5-ethylthio-1*H*-tetrazole solution (0.25 M) in acetonitrile for 12 min. Capping is done using acetic anhydride and 1-methylimidazole in tetrahydrofuran and pyridine. Oxidation was carried out using I_2_ solution in THF/Py/H_2_O (0.02 M). At the end, the synthesized products were cleaved from the solid support and deprotected with ammonium hydroxide solution and methylamine at 65 °C for 45 min. After drying the resulting RNA solution in a speed vacuum concentrator, RNA product was desilyated using 125 μL of triethylamine trihydrogen fluoride (Et_3_N·3HF) in 100 μL of dimethyl sulfoxide at 65 °C for 2.5 h. RNA was then precipitated by adding 0.025 mL of 3 M sodium acetate and 1 mL of cold absolute ethanol and stored at −80 °C for at least 1 h prior to centrifugation and drying under speed vacuum. The dried RNA product was reconstituted in an appropriate amount of water and obtained nanodrop reading for the concentration. Full length RNA templates were purified via gel extraction and validated the correct product on a 15% PAGE denaturing gel.

### Cloning of Native and Inosine-Modified RNA Strands

RNA templates were first reverse transcribed by each reverse transcriptase through primer extension to generate RNA/DNA hybrids. This was followed by lengthening the template by the addition of poly adenosine deoxynucleotides to the 3′hydroxyl terminus of the DNA strand using terminal transferase (TdT) (NEB M0315s). Subsequently, a set of primers was designed to bind to the polyadenylated tail and the other covering an adjacent region to amplify the sequence by PCR. Taq DNA polymerase was chosen in the interest of it lacking template-dependent and proofreading activities and for preferentially adding a single adenosine to the 3′end of the PCR amplicons to enable TA cloning. The linearized T-vector supplied in the TA cloning kit possesses an unpaired 3′ thymine residue complementary to the adenosine overhangs that can efficiently ligate with the PCR inserts to generate circular plasmid for downstream cloning into *Escherichia coli* competent cells. Cloning is achieved via transformation in chemically competent DH5α *E. coli*. The reverse transcription reactions are performed as described in the primer extension section above with the use of a non-FAM DNA primer. Enzyme was inactivated by heating to 65 °C for 20 min. For optimal A tailing, RNA templates were degraded in the RNA-DNA hybrid from the reverse transcription product by RNase H following the manufacture protocol (NEB M00297). DNA poly A tailing was performed in a final reaction volume of 50 μL containing 5 μL 10× TdT buffer, 250 μM CoCl_2_ (solution provided), 10 pmol of reverse transcription product from previous step, 10 nmol dATP, 10U of terminal transferase, and nuclease free water up to the final volume. According to the manufacturer’s recommendations, 1:1000 ratio of DNA to dATP is required for tail length extension of 10–20bp. The reaction was started by incubating at 37 °C for 30 min and terminated by heating it at 70 °C for 10 min. A tailing product was concentrated by speed vac for subsequent amplification by Taq polymerase. Briefly, we prepared the samples in a 25 μL reaction volume containing 5 μL of 5× OneTaq standard buffer, dNTPs (200 μM), forward primer 5′ A TCCCTGAGCGAC (0.2 μM), reverse primer 5′ TTTTTTTTTTTTTTTTTTTTTAGTCTG (0.2 μM), OneTaq DNA polymerase (0.625U), and nuclease free water if necessary. Thermal cycler perimeters: initial denaturation at 94 °C for 30 s, followed by 5 cycles of 94 °C 30 s, 43 °C 30 s, 60 °C 15 s, and 30 cycles of 94 °C 30 s, 45 °C 30 s, and 60 °C 15 s with 60 °C for 5 min as the final extension. Considering the low Tm value of our primers, we employed a combination of 5 cycles and 30 cycles.

Ligation of the DNA inserts with the pCR2.1 topo vector for cloning was performed in a 6 μL reaction volume as per manufacturer’s protocol: fresh PCR product (2 μL), salt solution provided (1 μL), water (2 μL), and topo vector (1 μL), incubated at 25 °C for 10 min followed by transformation into DH5α chemically competent *E. coli*. High efficiency transformation protocol was used according to the manufacture protocol for DH5α (NEB C2987H). The LB agar selection plates were precoated with kanamycin antibiotic at 50 μg/mL and 40 μL of 40 mg mL^–1^ of X-gal prior to spread the samples and incubated overnight at 37 °C. Light blue color colonies were picked, inoculated, and plasmids were extracted for Sanger sequencing.

### UV-Thermal Melting Temperature (*T*_m_) Study

Inosine-modified RNA with matched complementary DNA/RNA duplexes were prepared in sodium phosphate buffer containing 10 mM Na_2_HPO_4_, 10 mM NaH_2_PH_4_, and 100 mM NaCl at pH 7 by annealing 1.5 μM of purified RNAs with either complementary DNA/RNA strand in 600 μL total volume. The samples were heated at 95 °C for 5 min, slowly cooled down at RT for 2 h and incubated at 4 °C for another 2 h prior to measurement. Thermal melting temperature was determined by examining the absorbance versus temperature curves using a UV–vis spectrophotometer (Cary 300) equipped with a separate temperature controller. The reading was taken at the absorbance of 260 nM with total of four ramps collecting data points range from 5 to 85 °C at the rate of 0.5 °C/min. The temperature recorded was based on block temperature. Meltwin 3.5 software was used to obtain the thermodynamic parameters by analyzing the fitted melting curve of each duplex.

### Molecular Dynamics Simulations

To investigate the effect of inosine on the polymerase active site of reverse transcriptase, we simulated HIV-1-RT (PDB ID: IHYS)^47^ with the template RNA and product DNA strand. The structure of the RT was truncated by removing the RNase site to optimize the computational resources. First, AMBER^48^ type force–field parameters were developed for the inosine modification. The geometry of the modified nucleoside was optimized using the Hartree–Fock level theory and 6-31G* basis-sets. Partial charges on the atoms were then obtained using the online RESP charge-fitting server R.E.D.D.^49,50^ AMBER-99 force–field parameters with Chen–Garcia corrections were used for bonded and nonbonded interaction parameters for the modified nucleoside.^51^ Using MOE (Molecular Operating Environment) [https://www.chemcomp.com/Products.htm], the RNA/DNA sequence of the original structure was mutated to match that of the current experiment, and inosine modifications was modeled into the active site. Four simulations were performed where inosine in the substrate strand at the active site was paired with the four canonical nucleobases on the DNA strand-A,C,G, T.

Molecular dynamics simulations were performed using GROMACS 2019.4^52^ on all four systems in a solution of 0.1 M KCl in a cubic box. The size of the box and the number of ions and water molecules for the simulations were as follows: ∼13 nm containing 146 K^+^ and 143 Cl^–^ ions and ∼76,500 water molecules. The MD simulations incorporated a leapfrog algorithm with a 2 fs time step to integrate the equations of motion. The system was maintained at 300 K using the velocity rescaling thermostat.^53^ The pressure was maintained at 1 atm using the Berendsen barostat for equilibration.^54,55^ Long-range electrostatic interactions were calculated using the particle mesh Ewald algorithm with a real space cutoff of 1.0 nm.^56^ Lennard–Jones interactions were truncated at 1.0 nm. The TIP3P model was used to represent the water molecules, and the LINCS algorithm was used to constrain the motion of hydrogen atoms bonded to heavy atoms.^57^ The system was subjected to energy minimization to prevent any overlap of atoms, followed by a short equilibration (5 ns) and 50 ns production run. Coordinates of the RNA/DNA hybrid and protein were stored every 2 ps for further analysis. The simulations were visualized using Visual Molecular Dynamics (VMD) software^58^ and analyzed using tools from GROMACS.^52^ Hydrogen bonding analysis was performed in VMD using a donor–acceptor distance cutoff 0.33 nm and the hydrogen–donor–acceptor angle cutoff of 30°. All plots were generated in R and structure figures were generated in PyMOL (The PyMOL Molecular Graphics System, Version 2.0 Schrödinger, LLC. https://pymol.org/2/).

## Supporting Information Available

Cloning scheme for native and modified inosine RNA strands and sequencing logo representation of cloning results (PDF)
