# Supplementary material for: Inosine-Induced Base Pairing Diversity during Reverse Transcription
Source: ACS Chem Biol. 2024 Jan 22;19(2):348–56. doi: 10.1021/acschembio.3c00555 (PMC10877575; doi:10.1021/acschembio.3c00555)
Supplement: Supplementary file 1 — cb3c00555_si_001.pdf [file cb3c00555_si_001.pdf]

Supporting Information

Inosine Induced Base Pairing Diversity During Reverse Transcription

Ya Ying Zheng,[a,b] Kaalak Reddy,[b] Sweta Vangaveti,[b*] Jia Sheng[a,b,*]

[a]Department of Chemistry, and [b]The RNA Institute, University at Albany, State University of New York, 1400 Washington Avenue, Albany, NY 12222, USA
*Corresponding author: jsheng@albany.edu (J.S.), svangaveti@albany.edu (S.V.)

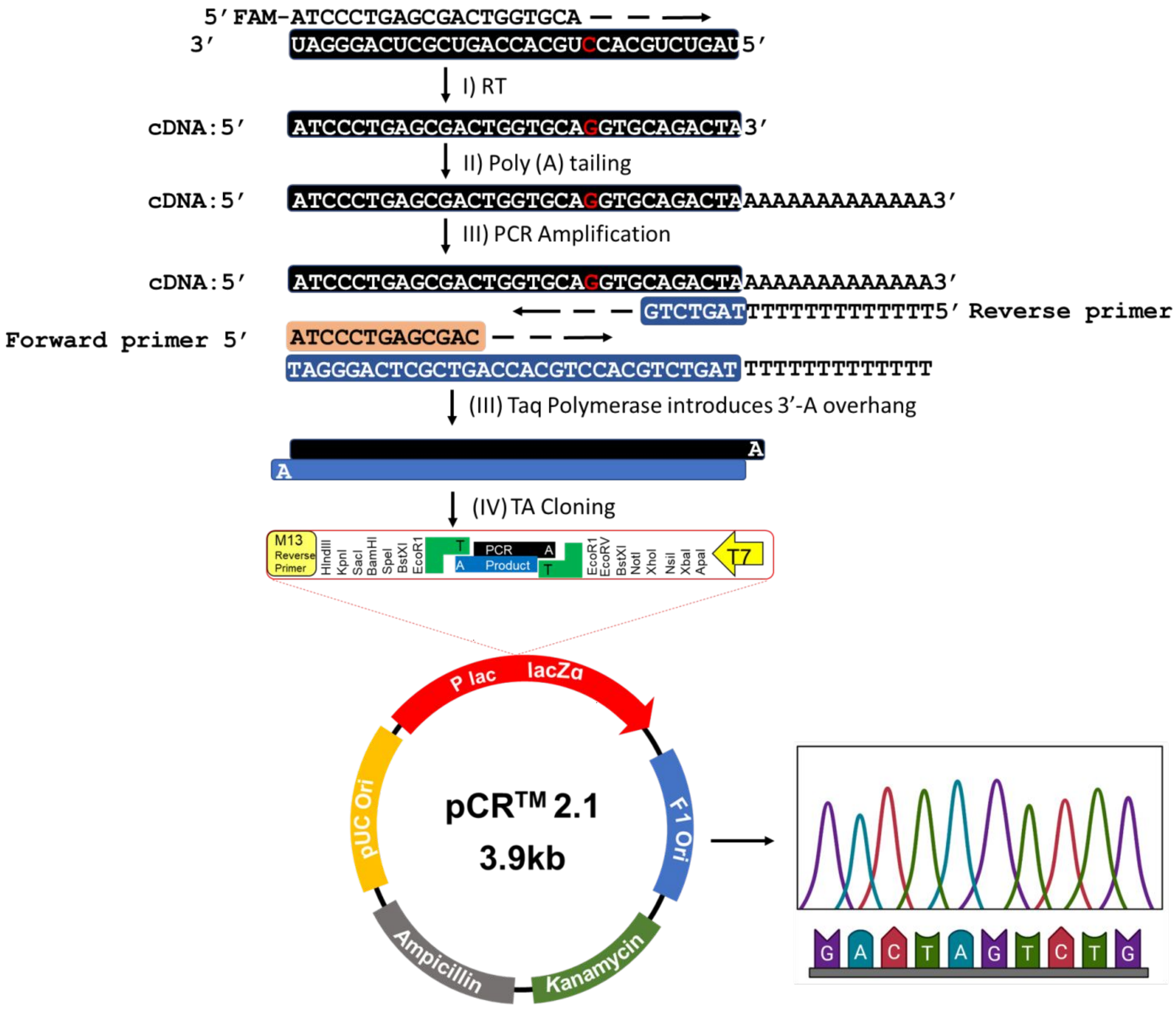

Figure S1. Cloning scheme for native and modified inosine RNA strands. RNA sequences were first converted into cDNA by means of primer extension utilizing various reverse transcriptases. cDNAs were subsequently poly adenylated followed by PCR amplification with regular forward primer and poly T reverse primer. The PCR amplicons were then liganded into TA vector. Following cloning procedure, the plasmids were extracted for standard Sanger sequencing.

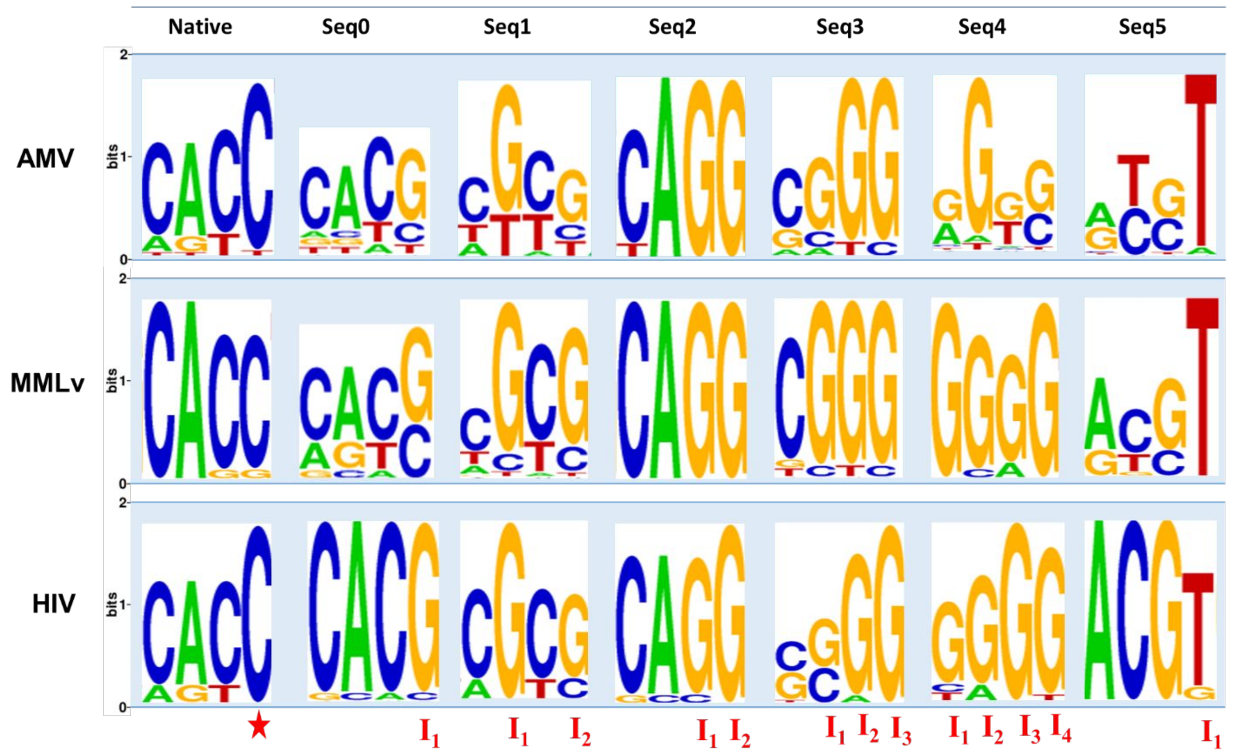

Table S1: Sequencing logo representation of cloning results. Consistent with the heatmap, the relative size of letter in the sequencing logo expressed the frequency of the most probable base readout. ★ is the native nucleotide in the unmodified RNA. Focusing just on modification sites in the sequence, I_1, I_2, I_3 and I_4 representing the position of inosine in the sequencing readout.
